# Variation in the mu-opioid receptor gene (*OPRM1*) moderates the influence of maternal sensitivity on child attachment

**DOI:** 10.1038/s41398-024-02888-x

**Published:** 2024-04-05

**Authors:** K. Tchalova, J. E. Lydon, L. Atkinson, A. S. Fleming, J. Kennedy, V. Lecompte, M. J. Meaney, E. Moss, K. A. O’Donnell, K. J. O’Donnell, P. P. Silveira, M. B. Sokolowski, M. Steiner, J. A. Bartz

**Affiliations:** 1 Department of Psychology, McGill University, Montréal, QC Canada; 2 Department of Psychology, Toronto Metropolitan University, Toronto, ON Canada; 3 Department of Psychology, University of Toronto Mississauga, Toronto, ON Canada; 4 Department of Psychiatry, University of Toronto, Toronto, ON Canada; 5 L’Université du Québec à Montréal, Montréal, QC Canada; 6 Douglas Research Centre, Montréal, QC Canada; 7 Singapore Institute for Clinical Sciences, A*STAR, Singapore, Singapore; 8 Yale School of Medicine, Yale University, New Haven, CT USA; 9 Department of Psychology, Simon Fraser University, Burnaby, BC Canada; 10 Department of Psychiatry, McGill University, Montréal, QC Canada; 11 Department of Ecology and Evolutionary Biology, University of Toronto, Toronto, ON Canada; 12 Department of Psychiatry and Behavioural Neurosciences, McMaster University, Hamilton, ON Canada

**Keywords:** Human behaviour, Neuroscience

## Abstract

The endogenous opioid system is thought to play an important role in mother-infant attachment. In infant rhesus macaques, variation in the μ-opioid receptor gene (*OPRM1*) is related to differences in attachment behavior that emerges following repeated separation from the mother; specifically, infants carrying at least one copy of the minor G allele of the *OPRM1* C77G polymorphism show heightened and more persistent separation distress, as well as a pattern of increased contact-seeking behavior directed towards the mother during reunions (at the expense of affiliation with other group members). Research in adult humans has also linked the minor G allele of the analogous *OPRM1* A118G polymorphism with greater interpersonal sensitivity. Adopting an interactionist approach, we examined whether *OPRM1* A118G genotype and maternal (in)sensitivity are associated with child attachment style, predicting that children carrying the G allele may be more likely to develop an ambivalent attachment pattern in response to less sensitive maternal care. The sample consisted of 191 mothers participating with their children (*n* = 223) in the Maternal Adversity, Vulnerability and Neurodevelopment (MAVAN) project, a community-based, birth cohort study of Canadian mothers and their children assessed longitudinally across the child’s development. Maternal sensitivity was coded from at-home mother-child interactions videotaped when the child was 18 months of age. Child attachment was assessed at 36 months using the Strange Situation paradigm. As predicted, G allele carriers, but not AA homozygotes, showed increasing odds of being classified as ambivalently attached with decreasing levels of maternal sensitivity. Paralleling earlier non-human animal research, this work provides support for the theory that endogenous opioids contribute to the expression of attachment behaviors in humans.

## Introduction

The key premise of attachment theory [1] is that children, as well as the young of other closely related species, are endowed with a biobehavioral *attachment system* that functions to keep them close to adults who can provide care and protection, thereby increasing the child’s chances of survival. Separation from the caregiver (called an *attachment figure* in the human research literature)—or cues signaling impending separation—evoke feelings of distress and set in motion behaviors like crying and searching to bring and keep the caregiver in close proximity. Once closeness to the caregiver has been restored, feelings of distress are replaced with feelings of comfort and contentment. A child (or other young primate) whose needs for security have been met is then able to engage with the world and pursue activities important for development, such as play and exploration; in this way, the attachment figure serves as a *secure base* for the child [2].

It is thought that this emotional bond between infants and their caregivers is rooted in evolutionarily ancient neural systems involved in processing pain and reward [3, 4]. The endogenous opioid system, which mediates the hedonic impacts of painful and rewarding stimuli [5, 6], has emerged as a likely neurochemical substrate for the potent affects that characterize attachment. Specifically, deprivation of contact with the mother is thought to induce a painful state analogous to opioid withdrawal, whereas reunion with the mother is thought to stimulate the endogenous opioid system, giving rise to feelings of pleasure and comfort, and relieving distress [7, 8]. Consistent with this proposition, morphine (an opioid receptor agonist), administered at low, non-sedative doses, is highly effective at alleviating separation distress in young animals [7, 9–13]. Conversely, naloxone or naltrexone (both opioid receptor antagonists) decrease behavioral indicators of comfort exhibited by animals in response to social contact [12–15]. Although work in humans is more limited, research indicates that naltrexone inhibits feelings of closeness and connection experienced in response to affectionate notes from loved ones [16] and photographs of close others [17]; moreover, the sadness participants experience while reflecting on memories of social loss is accompanied by de-activation of the μ-opioid system [18].

The attachment system is believed to be the genetically hardwired birthright of all species whose infants rely on caregivers for a prolonged period of time; however, individuals differ in their sensitivity to social separation (or cues signaling impending separation), and in their ability to gain reassurance from social contact and use caregivers as a secure base. This is true of both non-human animals and humans [19, 20]. A primary tenet of attachment theory is that the quality of caregiving children receive during development shapes the patterns of attachment-related affects and behaviors (collectively referred to as an *attachment orientation* or *style*) that an individual exhibits. In particular, attachment theorists have largely focused on maternal sensitivity as a major predictor of attachment styles. A history of sensitive parental care, wherein one’s bids for proximity and help are detected and met with reassurance, understanding, and warmth, is postulated to contribute to the emergence of attachment security. Attachment security is thought to reflect the “optimal” functioning of the attachment system: when threat (including distance from the attachment figure) is detected, the attachment system is activated, thus motivating proximity-seeking towards the attachment figure. Then, upon regaining proximity, a sense of security and comfort is restored, freeing the individual to engage in other pursuits. However, a relational history with a caregiver who has not been a consistent, predictable source of safety stymies reliance on the primary attachment strategy of proximity-seeking and results in the development of alternative, “secondary” attachment strategies [21] the child uses to meet their attachment needs.

These secondary attachment strategies take two forms. Some individuals may *deactivate* (or minimize) the attachment system, inhibiting attention to threats that may result in its activation, suppressing negative emotions, and emphasizing self-reliance. This is the avoidant phenotype. Other individuals may *hyperactivate* (or maximize) their attachment system. This hyperactivation manifests in heightened vigilance to attachment related cues and heightened emotional reactivity (anxiety, fear, anger) to threat, as well as intensified bids for closeness and reassurance (searching, crying)—sometimes continuing even after reassurance has been provided (e.g., clinging). This is the anxious/ambivalent phenotype. Essentially, deactivation and hyperactivation strategies are thought to arise in response to suboptimal caregiving environments. Of note, these different attachment strategies—or “styles”—have been extensively documented by Ainsworth [2] and others [22, 23] in studies using the Strange Situation paradigm to assess attachment in infants and young children.

While attachment is largely the product of one’s history of interpersonal interactions, genetically influenced differences in socioemotional processing may color perceptions of these interactions and influence emotional reactivity, thereby also contributing to differences in attachment in interaction with the social context [24]. Given the involvement of the endogenous opioid system in modulating emotional responses to social contact and separation [4, 15], genetic variation affecting components of this system may interact with the caregiving environment to shape attachment. Research in this area has focused on the functional μ-opioid receptor polymorphisms *OPRM1* A118G (in humans) and *OPRM1* C77G (in rhesus macaques), which share extensive similarities in terms of in vitro functional effects and behavioral phenotypes, and are thought to have arisen due to similar evolutionary pressures [25].

In a seminal study, Barr and colleagues ([26], see also [27]) investigated the role of *OPRM1* variation on attachment in rhesus macaques. In this study, they subjected the infants to four maternal separation-reunion cycles, with the aim of examining whether *OPRM1* variation differentially influences infant behavior in response to protracted periods of maternal unavailability. While all infants vocalized in distress immediately following the mother’s removal, G allele carriers cried more than CC homozygotes as the separation period continued. Further, while vocalizations declined among CC homozygotes over successive repetitions of the separation-reunion cycles, G allele carrying infants exhibited a potentiation of the separation-distress response, vocalizing at increasingly higher rates over repeated cycles. Moreover, unlike CC homozygotes, G allele carriers exhibited progressive increases in the amount of time they spent seeking contact with the mother upon reunion, at the cost of interacting with other group members. Thus, these data suggest progressive hyperactivation of the attachment system in response to repeated maternal separation among G allele carriers. While the functional consequences of this polymorphism are not entirely understood [25], it is worth noting that the effects of the minor G allele parallel the effects of opioid receptor blockade. That is, the attachment behaviors of G allele carrying infants in Barr et al. [26] study bear a striking resemblance to the attachment behaviors exhibited by rhesus macaque infants and juveniles who have received opioid receptor antagonists [14, 28]: specifically, more persistent elevations in distress vocalizations and stronger tendencies to cling to the mother and solicit her attention and care, while engaging in less play with their peers, suggesting an inability to be soothed. Together, these attachment behaviors are remarkably similar to the hyperactivated attachment behaviors exhibited by anxious/ambivalent human children.

Research in humans further suggests a link between the minor G allele and ambivalent attachment, or correlates of ambivalent attachment. Relative to AA homozygotes, adult participants carrying the minor G allele scored higher on trait rejection sensitivity (a prominent feature of anxious/ambivalent attachment) and exhibited stronger activation of pain-related neural regions during social exclusion [29, 30]. G allele-carrying mothers and their children were both more likely to self-categorize as insecurely attached (this study did not distinguish between anxiety and avoidance) than AA homozygotes [31]. The G allele was also associated with social withdrawal (a correlate of rejection sensitivity) in children [31, 32]. While these studies suggest that the minor G allele may predispose people to attachment system hyperactivation, the effects of this genotype should be considered in interaction with the caregiving environment, since *OPRM1* (and other genes involved in socioemotional processing) presumably exert their effects by serving as “filter” though which the environment is perceived, processed, and construed [24]. To this point, Bopari and colleagues [33] found that the G allele was related to separation anxiety disorder symptoms only among those children who displayed suboptimal interaction patterns with their mothers. Tchalova and colleagues [34] recently found further evidence for a gene by environment interaction effect for *OPRM1*. Specifically, in a three-week, intensive repeated measurement study of romantic couples, G allele carriers (vs. AA homozygotes) were more interpersonally reactive, exhibiting steeper declines in felt security in response to their partners’ self-reported quarrelsome behavior (e.g., criticism).

Thus, Tchalova et al. [34] and Bopari et al. [33] findings suggest that behavioral differences are produced by the combination of G allele carriers’ heightened interpersonal sensitivity and environmental cues like attachment figure non-responsiveness or potential signals of impending separation or rejection. That is, it is not simply the *OPRM1* A118G genotype but rather the *OPRM1* A118G genotype in combination with a specific caregiving environment (a “gene x environment” interaction) that should result in hyperactivated attachment behavior. Indeed, Barr et al. [26] also emphasized the importance of context in eliciting the effect of *OPRM1* variation. In their study, evidence of hyperactivated attachment strategies in G allele carrying monkey infants became evident following repeated bouts of separation from the mother. That is, there were no significant differences in the separation distress response on the first day separation; however, G allele carriers began to exhibit an increasing trajectory of hyperactivated attachment behavior with repeated, prolonged periods of maternal unavailability.

The main objective of the current research was to examine whether *OPRM1* similarly interacts with caregiving quality to produce differences in human attachment. To index the quality of the caregiving environment, we focused on maternal sensitivity, which, as described previously, refers to the extent to which the mother is able to understand, perceive, and appropriately respond to her child’s emotional and physical needs [35]. Lack of maternal sensitivity thwarts development of attachment security, leading to the development of secondary attachment strategies. Given the previous research, we predicted that G allele carries may be particularly likely to develop *hyperactivated* attachment strategies (i.e., attachment ambivalence) in response to low levels of maternal sensitivity. To test this hypothesis, we drew upon data from the Maternal Adversity, Vulnerability and Neurodevelopment (MAVAN) project [36], which includes longitudinal behavioral assessments of maternal sensitivity and child attachment assessed with the Strange Situation procedure.

## Methods

### Participants

Sample characteristics are presented in Table 1. The current sample consisted of 191 mothers and their children (*n* = 223) who participated in the MAVAN project, a large community-based birth cohort study of Canadian mothers and their children (see [36] for a detailed description of the project’s scope and methods). Pregnant women were recruited at 13 to 25 weeks’ gestation from hospital obstetric clinics in Montréal, Québec and Hamilton, Ontario. To be eligible, women were required to have singleton pregnancies, be 18 years of age or older, and be fluent in either English or French. Exclusion criteria included serious obstetric complications, extremely low birth weight, prematurity (≤37 weeks’ gestation), or presence of congenital disease or defect. Of the 191 mothers enrolled in this study, 160 participated with one child, 30 participated with two children, and one participated with three children. Ethics approval for the study was obtained from the Douglas Mental Health University Institute (Montréal) and St. Joseph’s Hospital (Hamilton).

**Table 1:** Sample characteristics.

	Avoidant (*n* = 13)	Secure (*n* = 129)	Ambivalent (*n* = 32)	Disorganized (*n* = 49)
Sex of child (% female within category)	30.8	51.2	50.0	38.8
Genotype (% G allele carriers within category)	30.8	26.4	25.0	16.3
Birth weight (average in grams)	3511.5	3367.2	3418.1	3352.3
Gestational age (average in weeks)	39.1	39.4	39.5	39.6
Maternal age at delivery (average in years)	31.8	31	31.7	29.3
Mother’s education level (%)				
High school or less	38.5	14.7	12.5	30.6
College	23.1	28.0	28.1	24.5
University degree	38.5	57.4	59.4	44.9
Household income (% within category)				
Between $0 and $20,000	7.7	7.0	3.1	12.2
Between $20,000 and $40,000	15.4	10.9	18.8	8.2
Between $40,000 and $60,000	15.4	10.1	12.5	18.4
Between $60,000 and $80,000	38.5	16.3	9.4	14.3
Between $80,000 and $100,000	7.7	19.4	25.0	12.2
$100,000 or more	7.7	17.8	18.8	0.0
Not available	7.7	18.6	12.5	34.7
Maternal sensitivity at 18 mos (average; scale range 1–9)	6.7	6.3	5.7	5.6
Maternal depression at 36 mos (average; scale range 0–60)	12.8	10.2	13.7	13.8
Maternal anxiety at 24 mos (average; scale range 20–80)	36.2	35.4	36.1	39.4
Maternal perceived stress at 36 mos (average; scale range 0–4)	1.2	1.1	1.2	1.3
Maternal marital stress at 36 mos (average; scale range 1–7)	2.7	2.4	2.8	2.8

Thirty-one mothers participated in the study with more than one child and therefore provided data more than once.

Socioeconomic data were collected during prenatal visit.

Two records were missing for maternal depression, 25 for maternal anxiety, and 18 for marital stress.

### Measures

#### Maternal sensitivity

Maternal sensitivity was coded from mother-child interactions videotaped during an at-home visit that took place when the child was 18 months old. During this visit, the mother was asked to interact with her child for 30 minutes. This interaction consisted of four phases: (1) free-play (5 min); (2) play without any toys (10 min); (3) play with a standardized assortment of toys brought by the research assistant (10 min); and (4) completion of questionnaires without play (intended to assess maternal sensitivity when the mother’s attention is divided; 5 min). These videotaped interactions were subsequently coded using the Ainsworth Maternal Sensitivity scales [35], an extensively validated and widely used measure of maternal sensitivity [2, 23, 37]. Specifically, the Ainsworth scales are a macro-analytic measure where four key aspects of maternal care—sensitivity to child signals, cooperation vs. interference with child activities, psychological and physical availability, and acceptance vs. rejection of child needs—are rated on 9-point scales, with higher scores reflecting more sensitive care. All four scales have been shown to differentiate mothers of secure infants from mothers of both avoidant and ambivalent children [2]; because the subscales tend to be highly correlated (*r*s ranging from 0.86 to 0.92 in the present sample), we averaged them into one maternal sensitivity score as in prior research (e.g., [38]). Inter-rater reliability was high, ICC = 0.92 (*n* = 18).

#### Child attachment

Child attachment was assessed during an in-lab visit at 36 months using the Preschool Separation-Reunion Procedure [39], a modified version of the Strange Situation [2] developmentally suitable for use with preschool-aged children (see [40–42] for validation studies). This procedure consists of a 20-minute-long assessment period comprising a sequence of separations/reunions lasting 5 minutes each: (1) separation between mother and child; (2) reunion; (3) second separation, and (4) second reunion. Following standard procedure, the child’s behavior (physical contact and/or seeking or maintenance of physical proximity, body position, content and style of speech directed toward the mother, looking behavior directed toward the mother, verbal and non-verbal indices of affect) was coded by trained observers from videotape using the MacArthur Preschool Attachment Coding System (PACS) [39], giving rise to one of four attachment classifications: secure, ambivalent, avoidant, or disorganized [43]. In this coding scheme, a secure attachment style is indicated by the child’s effective use of the mother as a secure base for exploration, as well as relaxed, mutually enjoyable interaction between mother and child. Contrastingly, ambivalent attachment is characterized by reduced exploration as well as excessive preoccupation with the mother (e.g., following her around the room, asking to be held), accompanied by heightened distress. Avoidant attachment is marked by avoidance of intimate interaction with the mother. Finally, children who appear to lack a coherent attachment strategy are classified as disorganized. In this sample, there were 129 secure, 32 ambivalent, 13 avoidant, and 49 disorganized classified children. [Note that the percentage of children classified as disorganized is consistent with the rates reported in two recent large meta-analyses of children in pre-school years [44] and infancy [45] (21.5% and 23.5%, respectively).]

In addition to generating the classic categorical attachment classifications with the PACS, coders also rated the children’s attachment behavior on 9-point security, ambivalence, avoidance, and disorganization dimensions using the Preschool Attachment Rating Scales (PARS) [46, 47]. [PARS data were not available for one child.] Of note, inspection of the data revealed several children with a primary PACS classification of secure or disorganized also scored high in ambivalence on the PARS (3 and 15 children, respectively). To capture these high ambivalence scorers, we devised an additional classification system in which we utilized the continuous ambivalence score to create a new dichotomized variable, with children scoring below 5 being classified as low ambivalence and children scoring 5 or above being classified as high ambivalence (this threshold was selected based on guidelines outlined in the PARS manual [47]). Using this new categorical classification along with the original categorical classifications also allowed us to confirm the robustness of any findings from the original categorical classifications with the advantage of a slightly increased sample of ambivalent children (i.e., with this new classification, the high ambivalent group increased from 32 to 49 children).

#### Genotyping

Buccal epithelial cells were collected from each participant (at 36-months) and genotypes were determined via genome-wide platforms (PsychArray/PsychChip, Illumina) using 200 ng of genomic DNA according to manufacturer’s guidelines. Quality control procedure was carried out using PLINK 1.9 [48]. Samples with a call rate less than 90% were removed. Exclusion criteria included: SNPs with a low call rate (<95%), low p-values on Hardy–Weinberg Equilibrium (HWE) exact test (*p* < 1e–40), and low minor allele frequency (MAF) (<5%). We performed imputation using the Sanger Imputation Service [49] and the Haplotype Reference Consortium (HRC) as the reference panel (release 1.1). *OPRM1* A118G polymorphism (rs1799971) was then extracted from the imputed data, having an info score = 1.0.

Among the 223 children used in the present analyses, 169 were AA homozygotes, 51 were AG heterozygotes, and 3 were GG homozygotes. Using Haldane’s exact test implemented in the Hardy–Weinberg package [50] in R (version 4.0.3), we established that the observed genotype frequencies did not deviate from those expected under Hardy–Weinberg equilibrium, *D* = 0.64, *p* = 1.00. Due to the small number of GG homozygotes, the AG and GG groups were combined, as has been done in prior research [29].

### Analysis plan

Our primary hypothesis was that child genotype would interact with maternal sensitivity at 18 months to predict child attachment classification as assessed in the Strange Situation paradigm at 36 months. Specifically, we predicted that children with the minor G allele would be more sensitive to variation in maternal care and, consequently, more likely to be categorized as ambivalent (vs. secure) than those homozygous for the A allele in response to maternal insensitivity. To test our hypotheses, we conducted a multinomial logistic regression analysis with child genotype (effect coded as −1 = AA homozygote; 1 = G allele carrier), maternal sensitivity (grand-mean centered), and the child genotype by maternal sensitivity interaction predicting the likelihood of being categorized as ambivalent (vs. secure) at 36 months. While our primary hypothesis concerned ambivalence, given prior links between *OPRM1* variation and interpersonal sensitivity and parallels with non-human animal research, our statistical model also tested the likelihood of being classified as either avoidant or disorganized (vs. secure). Analyses were carried out in Mplus 8 [51]. For all analyses, we used Maximum Likelihood estimation with robust standard errors (MLR) and the CLUSTER function to account for the non-independence of observations arising from children clustered within families by producing correctly adjusted standard errors [51].

As noted, in assigning children to attachment categories, we followed the standard PACS procedure but also devised an additional classification system using the PARS, which allowed us to capture children who scored high in ambivalence even though that was not their initial classification. This allowed us to test the robustness of the effects. We summarize results for both approaches below. [As is typical in developmental attachment research, we focused on predicting attachment classification. However, one could also examine the continuous attachment outcomes; for the interested reader, we present these alternative analyses in the Supplementary Material. Note that the continuous analyses do not entirely parallel the categorical analyses; we discuss possible reasons for this in the Supplementary Material]

## Results

Results of the multinomial logistic regression predicting attachment classification at 36 months as a function of child genotype, maternal sensitivity at 18 months, and the child genotype by maternal sensitivity interaction are reported in Table 2.

**Table 2:** Results of multinomial logistic regression predicting attachment classification at 36 months (reference group: secure) and binomial logistic regressions predicting high vs. low scores on ambivalent, avoidant, and disorganized attachment dimensions as a function of child genotype and maternal sensitivity at 18 months.

	B(SE)	*p*	odds ratio	95% CI		B(SE)	*p*	odds ratio	95% CI
Ambivalence vs. security					High vs. low ambivalence				
Intercept	−1.57 (0.28)	<0.001	NA	NA	Intercept	−1.42 (0.24)	<0.001	NA	NA
Child genotype	−0.20 (0.26)	0.452	0.82	[0.49, 1.37]	Child genotype	−0.23 (0.23)	0.316	0.79	[0.50, 1.25]
** Maternal sensitivity**	**−0.46 (0.17)**	**0.007**	**0.63**	**[0.45, 0.88]**	**Maternal sensitivity**	**−0.45 (0.16)**	**0.004**	**0.64**	**[0.47, 0.87]**
**Child genotype x maternal sensitivity**	**−0.38 (0.17)**	**0.026**	**0.68**	**[0.49, 0.95]**	**Child genotype x maternal sensitivity**	**−0.42 (0.16)**	**0.007**	**0.65**	**[0.48, 0.89]**
Avoidance vs. security					High vs. low avoidance				
Intercept	−3.40 (0.60)	<0.001	NA	NA	Intercept	−2.19 (0.30)	<0.001	NA	NA
Child genotype	−1.04 (0.60)	0.082	0.35	[0.11, 1.14]	Child genotype	0.09 (0.30)	0.762	1.09	[0.61, 1.95]
** Maternal sensitivity**	**0.77 (0.30)**	**0.011**	**2.15**	**[1.20, 3.87]**	**Maternal sensitivity**	**0.25 (0.17)**	**0.141**	**1.28**	**[0.92, 1.79]**
** Child genotype x maternal sensitivity**	**0.82 (0.30)**	**0.006**	**2.27**	**[1.26, 4.07]**	**Child genotype x maternal sensitivity**	**0.38 (0.17)**	**0.024**	**1.47**	**[1.05, 2.04]**
Disorganization vs. security					High vs. low disorganization				
Intercept	−1.20 (0.26)	<0.001	NA	NA	Intercept	−1.61 (0.26)	<0.001	NA	NA
Child genotype	−0.28 (0.26)	0.297	0.76	[0.45, 1.27]	Child genotype	−0.16 (0.25)	0.516	0.85	[0.52, 1.39]
Maternal sensitivity	−0.12 (0.15)	0.403	0.89	[0.66, 1.19]	Maternal sensitivity	−0.10 (0.11)	0.391	0.91	[0.73, 1.13]
Child genotype x maternal sensitivity	0.18 (0.15)	0.238	1.19	[0.89, 160]	Child genotype x maternal sensitivity	0.15 (0.11)	0.180	1.16	[0.93, 1.44]

### Child OPRM1, maternal sensitivity & attachment ambivalence (vs. security)

**Fig. 1:**
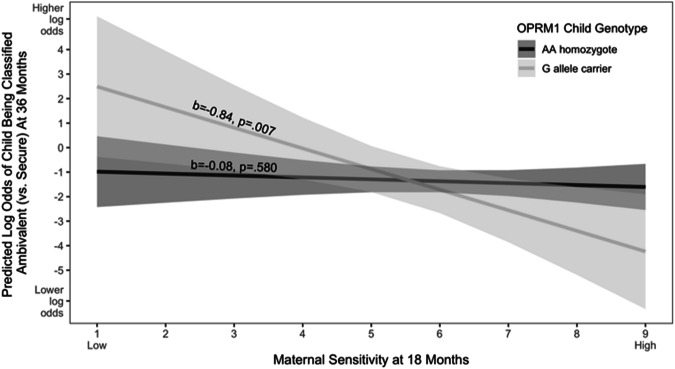
Results of multinomial logistic model predicting classification as ambivalent vs. secure as a function of OPRM1 child genotype and maternal sensitivity. The *X*-axis reflects levels of maternal sensitivity measured when the child was 18-months old, with higher numbers indicating greater maternal sensitivity. The *Y*-axis reflects the predicted log odds of the child being classified as ambivalent (vs. secure) at 36-months, with higher numbers indicating greater log odds of being classified as ambivalent (vs.secure). Shaded areas represent 95% confidence intervals.

Results of the multinomial logistic regression predicting the log odds of being classified as ambivalent (vs. secure) revealed an overall main effect of maternal sensitivity, such that lower levels of sensitivity predicted higher log odds of being classified as ambivalent (vs. secure), *b* = −0.46, *p* = 0.007. Critically, however, as predicted, results also revealed a child genotype by maternal sensitivity interaction, *b* = −0.38, *p* = 0.026 (Fig. 1), such that the effect of maternal (in)sensitivity was evident among G-allele carriers, *b* = −0.84, *p* = 0.007 but not AA homozygotes, *b* = −0.08, *p* = 0.580.

Using our alternate parameterization of attachment ambivalence (high vs. low), we similarly obtained a significant main effect of maternal sensitivity, *b* = −0.45, *p* = 0.004, as well as a significant child genotype by maternal sensitivity interaction, *b* = −0.42, *p* = 0.007. Consistent with our earlier finding, maternal sensitivity was negatively related to the log odds of scoring high (vs. low) on attachment ambivalence for G allele carriers, *b* = −0.87, *p* = 0.003, whereas it did not have a significant relationship with attachment ambivalence among AA homozygotes, *b* = −0.02, *p* = 0.846.

### Child OPRM1, maternal sensitivity & other attachment classifications

Turning to the other insecure attachment categories, results revealed an overall main effect of maternal sensitivity on the avoidance classification. Specifically, maternal sensitivity at 18 months positively predicted higher log odds of being classified as avoidant vs. secure at 36 months, *b* = 0.77, *p* = 0.011. Results also revealed a child genotype by maternal sensitivity interaction, *b* = 0.82, *p* = 0.006, indicating that the positive association between maternal sensitivity and the log odds of being classified as avoidant (vs. secure) was driven by the G allele carrying children, *b* = 1.59, *p* = 0.005 (there was no relationship between maternal sensitivity and the log odds of being classified avoidant in the AA genotype group, *b* = −0.05, *p* = 0.779). Using our alternate parameterization of avoidance, there was no longer a significant effect of maternal sensitivity on avoidance *b* = 0.25, *p* = 0.141; however, the child genotype by maternal sensitivity interaction held, *b* = 0.38, *p* = 0.024. Given that avoidance is characterized by attachment system *de*activation, this interaction effect is in line with our overall prediction that, in the presence of maternal insensitivity, the G allele is associated with hyperactivated attachment behavior. Essentially, G allele carrying children are especially *un*likely to use deactivation strategies in response to maternal insensitivity. That said, we are reluctant to give much weight to this observation given that there were very few children in the avoidant G carrier group (*n* = 13) and that the range of maternal sensitivity scores in this group was highly restricted.

Finally, there were no significant effects of either maternal sensitivity, *b* = −0.12, *p* = 0.403, or child genotype, *b* = −0.28, *p* = 0.297, on the log odds of being classified as disorganized (vs. secure), nor was there a significant interaction between maternal sensitivity and child genotype, *b* = 0.18, *p* = 0.238. Neither were there any significant effects using our alternate parameterization of disorganized attachment (see Table 2). [Note: maternal sensitivity on its own (i.e., without genotype of the maternal sensitivity by genotype interaction in the model) did negatively predict the log odds of being classified as disorganized (vs. secure), *b*  = −0.26, *p*  = 0.009.].

## Discussion

We investigated the interactive effects of maternal sensitivity and child *OPRM1* A118G genotype on child attachment behavior. We predicted that the more interpersonally sensitive G allele carriers would be more likely than the AA homozygotes to respond to maternal insensitivity with attachment ambivalence. As noted at the outset, attachment insecurity in humans is not a homogenous construct; rather, it can take the form of ambivalence or avoidance. That is, in response to an insensitive caregiver, infants and children cope by either (1) hyperactivating the attachment system—ramping up behaviors like crying, searching, and clinging to secure the elusive caregiver’s attention—or (2) deactivating the attachment system—dampening down such attachment related behaviors. Our data indicate that the *OPRM1* 118G allele predisposes children to the former strategy. This finding parallels earlier work in non-human primates showing an association between an analogous *OPRM1* genetic variant and displays of hyperactivated attachment behavior in response to repeated maternal separation [26]. That is, both macaque infants and human children carrying these genetic variants exhibit similar patterns of heightened and more persistent separation distress (crying), clinging to their mothers, and disinterest in exploration (i.e., inability to use the mother as a secure base), particularly after being subjected to inconsistent maternal care. This research is also consistent with other work in humans linking this genetic variant to greater rejection sensitivity [29], separation anxiety disorder [33], and attachment insecurity [31, 32]. However, the current work represents an important advance over earlier research on *OPRM1* and attachment in humans by being the only study to use extensively validated, objective behavioral measures of both maternal sensitivity and child attachment, as well as longitudinal assessment, thereby increasing reliability, generalizability, and strengthening causal claims.

We would like to highlight that we did not find evidence for main effect of genotype—rather, our findings reflect a gene by environment interaction. That is, G allele carriers were more likely to be categorized as ambivalent when their mothers displayed lower maternal sensitivity. Intriguingly, in addition to finding that G allele carriers were more likely to be categorized as ambivalent (vs. secure) when their mothers were less sensitive, we also found that G allele carriers were *less* likely to be categorized as ambivalent (vs. secure) when their mothers were *more* sensitive (see Figure S4 in the Supplementary Online Materials for a visualization of this effect). That is, in the presence of high levels of maternal sensitivity, G allele carriers fared better than their AA counterparts. Although this observation warrants replication, it is consistent with theorizing and empirical work on differential susceptibility to environmental influences [52] and biological sensitivity to context models [53]. In essence, rather than being a risk factor (c.f., stress-diathesis models), such reactivity makes these individuals more sensitive to all experiences—negative and positive. Consistent with this idea, in the aforementioned study by Tchalova et al. [34] assessing individuals’ feelings of security in response to their romantic partner’s cold/quarrelsome behavior, the largest discrepancy in felt security between the G allele carriers and the AA homozygotes was evident at the low end of partner quarrelsome behavior. That is, G allele carriers reported *higher* levels of felt security than their AA counterparts when their partner behaved *less* quarrelsomely than usual. Although the absence of a negative behavior (e.g., fewer criticisms) is not the same as a positive behavior (e.g., more affection), these data also suggest increased sensitivity to various kinds of social input (not just threat). Further, this finding is also consistent with research demonstrating that relief experienced due to the omission or attenuation of an expected aversive event is experienced as pleasurable, and that the pleasantness of the relief is related to the degree of negative expectation [54]. That is, to the extent that pleasure from relief stems from a violation of a negative expectation, individuals who hold more negative expectations may experience more reward when these expectations are violated. While this has been demonstrated for a general dispositional tendency toward negative expectations (i.e., pessimism; [54]), it may also be the case for individuals who hold negative relational expectations, like the anxious-ambivalent children in our study. Thus, it is possible that G allele carriers may be more sensitive to socially painful experiences, but also prone to experiencing a greater degree of relief when social interactions unfold better than expected. [We thank an anonymous reviewer for the suggestion that expectancy violation dynamics may help explain some of the findings for G allele carriers.]

A related possibility is that G allele carriers experience greater reward from positive social interactions. Indeed, Troisi et al. [55] found that G allele carriers reported greater engagement in and pleasure from social relationships compared to their AA counterparts. However, such reward sensitivity makes them especially distressed when there is a threat to their interpersonal relationships and more motivated to re-establish connection. Consistent with this, Copeland and colleagues [56] found that older children (9–17-year-olds) carrying the G allele, who came from home environments characterized by parental dysfunction (e.g., mental health problems, substance abuse, criminality), reported greater enjoyment of interactions with their parents and fewer parent-child arguments than the AA homozygotes. Such behaviors are consistent with the ambivalent profile [57]—that is, idealizing the caregiver/relationship with the caregiver (at least some of the time), responding to poor or inconsistent care by increasing attempts to gain closeness to the caregiver, and efforts to appease the caregiver (e.g., with fewer arguments). It remains to be seen whether the G allele confers greater sensitivity to various kinds of social input but, taken together with our data, it appears that these G carrying children do not give up—they persist in their hyperactivating strategies, even at age 9–17 years, to connect with elusive caregivers.

One interesting question for future research concerns the role of *OPRM1* variation in the context of more enduring separations, such as, for example, those arising from parental divorce, illness, death, or, in adulthood, relationship dissolution. Would G allele carriers be at higher risk of negative outcomes following the loss of attachment relationship? Although amplified attempts to gain social support may be an adaptive strategy for eking out support from a reluctant or distracted caregiver in childhood (see Barr et al. [26], for discussion of the potential evolutionary value of this variant in adverse environmental conditions, such as those characterized by resource scarcity), such hyperactivated seeking could prove detrimental when support is lost forever (see [58] for a relevant discussion of the role *OPRM1* and endogenous opioids may play in recovery following bereavement).

Some important limitations of the present research, along with opportunities for further research, should be acknowledged. Due to the methodological complexities involved in conducting a longitudinal study of mother-infant dyads with behavioral assessments, the overall sample size is small for a genetic study; further, only a subset of children was categorized as ambivalent. These two factors limit our statistical power. Thus, the current findings should be considered preliminary prior to replication. That said, while statistical power is a function of sample size, another key determinant of statistical power is measurement precision [59]. To the extent that direct observational measures of maternal sensitivity and child attachment entail less measurement error than self- or parental-reports, this feature of the current design should be considered a countervailing factor boosting statistical power. A second limitation is that the MAVAN sample is a predominantly Caucasian sample recruited from two Canadian provinces (Québec and Ontario). Thus, generalizability of these findings to other populations must be established. Finally, we focused on a single candidate polymorphism in a complex system; further research is required to examine the contribution of *OPRM1* A118G relative to, and in interaction with, other genetic variants in shaping attachment ambivalence in relation to maternal sensitivity. That said, some concerns regarding the SNP approach should be alleviated in part by (1) the empirically supported theory behind selection of the *OPRM1* A118G SNP, (2) converging findings across not only independent studies and populations in humans, but also (3) recapitulation of an association previously observed in an animal model [26], and (4) evidence that this SNP has functional consequences for μ-opioid receptor expression and activity [60].

In addition to examining the μ-opioid system in interaction with other neurochemical systems believed to play an important role in attachment processes (e.g., oxytocin, dopamine [61]), future research in this area would also benefit from consideration of epigenetic processes and genetic-epigenetic interactions in relation to differences in attachment experiences. To this point, the new CpG methylation site introduced by the A118G nucleotide exchange has been found to affect methylation patterns and, consequently, structural changes in the μ-opioid system in response to prolonged opioid use [62], and similar genetic-epigenetic interactions have been found to shape stress reactivity in response to maternal deprivation in non-human animals [63].

## Summary

In sum, consistent with theory and decades of prior research on attachment, we found that variation in maternal sensitivity at 18 months significantly predicted the likelihood that the child would be classified as ambivalent vs. secure at 36 months of age. Critically, however, we also found support for our prediction that the effect of maternal sensitivity on child ambivalence would be moderated by the child’s *OPRM1* genotype, with the G allele carrying children being significantly more likely to be classified as ambivalent in response to maternal insensitivity. Thus, consistent with earlier non-human animal research, this finding suggests that carrying the G allele predisposes the child to a specific form of insecurity in the face of inconsistent maternal care—one characterized by a heightened emotional reaction to separation, a preoccupation with the mother, and an inability to be soothed by the mother’s return. More broadly, these findings provide support for the brain opioid theory of social attachment [64], which postulates that changes in endogenous opioid activity in response to social separation and social contact mediate the affective experiences of separation distress and social comfort, respectively, and thereby contribute to the regulation of attachment behavior.

### Supplementary information

Supplementary Online Materials for Variation in the mu-opioid receptor gene (OPRM1) moderates the influence of maternal sensitivity on child attachment

## Data Availability

The data for this study will be shared upon request to the corresponding author.
