# Supplementary material for: Variation in the mu-opioid receptor gene (OPRM1) moderates the influence of maternal sensitivity on child attachment
Source: Transl Psychiatry. 2024 Apr 5;14:181. doi: 10.1038/s41398-024-02888-x (PMC10997775; doi:10.1038/s41398-024-02888-x)
Supplement: Supplementary file 1 — Supplementary Online Materials for Variation in the mu-opioid receptor gene (OPRM1) moderates the influence of maternal sensitivity on child attachment [file 41398_2024_2888_MOESM1_ESM.docx]

Supplementary Online Materials for

**Variation in the mu-opioid receptor gene (*OPRM1*) moderates the influence of maternal sensitivity on child attachment**

**This document includes:**

1. Analyses Investigating the Effects of Child *OPRM1* genotype and Maternal Sensitivity on Continuous Attachment Ambivalence Scores
2. Figure S1: Effect of Child *OPRM1* genotype and Maternal Sensitivity on Continuous Ambivalence Scores
3. Categorical and Continuous Attachment
4. Figure S2: Distribution of Children’s Continuous Attachment Scores for Each Attachment Classification Group
5. Figure S3: Comparison of Children’s Categorical Attachment Labels with Their Scores on the Continuous Attachment Dimensions
6. Johnson-Neyman Analysis Probing Child *OPRM1* Genotype by Maternal Sensitivity Interaction Predicting Child Ambivalence
7. Figure S4: Johnson-Neyman Plot Depicting Regions of Significance for the Child *OPRM1* Genotype by Maternal Sensitivity Interaction Predicting Child Ambivalence

**Analyses Investigating the Effect of Child *OPRM1* genotype and Maternal Sensitivity on Continuous Attachment Ambivalence Scores**

Child attachment has historically been assessed with a categorical measure and this continues to be the dominant approach used today. That said, researchers have also used ratings from the continuous dimensions of security, ambivalence, avoidance, and disorganization to assess attachment (1,2). Although our initial focus was on the categorical data (as is standard in the field), we were curious whether the results could be replicated with the continuous data, and report those analyses here in the interest of transparency. Here we focus on the main outcome of interest—ambivalence—but report analyses for the other dimensions in a footnote for interested readers.

Few studies have used the PARS as an outcome so there is no standardized data analytic approach. Thus, we first ran a multiple regression analysis with child *OPRM1* genotype, maternal sensitivity at 18 months, and the child *OPRM1* genotype by maternal sensitivity interaction as predictors of the continuous ambivalence scores. As with the categorical analyses, there was a significant effect of maternal sensitivity on child ambivalence, *b*=-0.22, *p*=.012 (95% CI: -0.40, -0.05), such that mothers who were rated as more sensitive at 18 months had children who were rated as less ambivalent at 36 months. However, there was no main effect of child genotype, *b*=0.02, *p*=.900, or interaction between child genotype and maternal sensitivity, *b*=-0.04, *p*=.701.^[[1]](#footnote-1)^

Of note, one difference between the multinomial logistic regression analysis of the categorical measure and the linear regression analysis of the continuous measure is that the key contrast of interest in the former analysis compared those with a primary diagnosis of ambivalent vs. secure and does not include those with a diagnosis of avoidance or disorganization. By contrast, the linear regression analysis of the continuous attachment measure includes all participants, which makes this a much more heterogenous group. Thus, it may be important to control for where kids stand on the other attachment dimensions in our analyses. To this end, we ran a second regression analysis, identical to that described above, but with the inclusion of the continuous security, avoidant and disorganization measures as covariates. Although the interaction between child genotype and maternal sensitivity did not reach statistical significance, *b*=0.19, *p*=.085, results parallel the logistic regression analyses reported in the main text. Specifically, as depicted in **Figure S1**, maternal sensitivity was negatively related to ambivalence scores of G allele carrying children, *b*=-0.22, *p*=.022, but not the ambivalence scores of AA homozygotes, *b=*-0.03, *p*=.670.^[[2]](#footnote-2)^

**Figure S1.** Effect of Child *OPRM1* genotype and Maternal Sensitivity on Continuous Ambivalence Scores


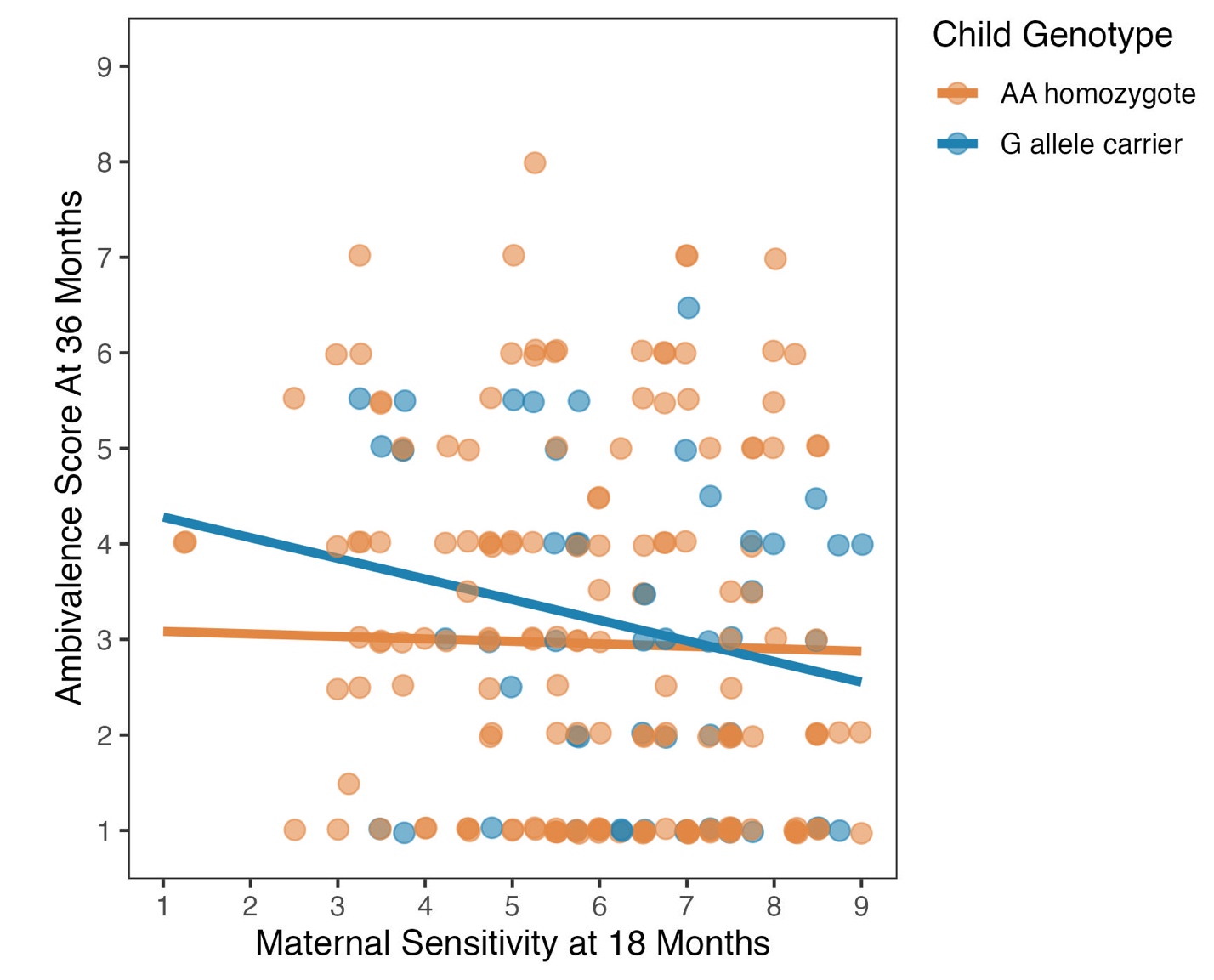


In sum, our attempt to replicate the categorical analyses using the continuous outcomes was mixed. Results parallel the categorical approach—with maternal sensitivity being associated with child ambivalence for G allele carriers but not for AA homozygotes—although the effect fell short of statistical significance, and it was important to control for the other attachment dimensions in the analysis. This latter point is, perhaps, not surprising when one considers the variance introduced by including children with a primary diagnosis of avoidance or disorganization (see Categorical and Continuous Attachment and Figure S3 for details). Indeed, the categorical approach is, to date, the standard approach taken in most studies of attachment in children.

**Categorical and Continuous Attachment**

The MacArthur Preschool Attachment Coding System (PACS) (3) assess attachment by categorizing children into one of four attachment classifications: secure, ambivalent, avoidant, or disorganized (4). In addition, children’s behavior can be rated on the Preschool Attachment Rating Scales (PARS) (1,2), which yields continuous security, ambivalence, avoidance, and disorganization scores for each child. The PACS assesses the predominant strategy used by the child, whereas the PARS focuses on the nature of the range of behaviors displayed by the child during the Strange Situation paradigm. Essentially, the PACS is the balance of one set of behaviors over others that define the classification. Both secure and ambivalent children share an orientation toward the caregiver. What differentiates them is that secure children balance this orientation with exploration of the environment, while ambivalent children show a more exclusive preoccupation with the caregiver. As depicted in **Figure S2**, there is consistency between the categorical classifications and continuous scores—i.e., children categorized as secure have the highest continuous security scores, children categorized as ambivalent have the highest continuous ambivalent scores, etc.

That said, there is also variation. In the example above, the child’s overall classification would be ambivalent (because of preoccupation with mom), but the child also displays some behaviors indicative of security (orientation to mom) and thus would have a moderately high security score on the PARS. In Figure S3, we overlay the attachment classification on the continuous dimension. Taking a look at the security dimension (**Figure S3, upper left**), one can see that the ambivalent group is not low in security; rather they are at the mid-point (hence ambivalence, and example above), whereas the avoidant and disorganized groups are low. Taking a look at the continuous ambivalence dimension (**Figure S3, lower left**), one can see that there is a good number of children categorized as disorganized with high ambivalence scores (there are also many with low ambivalence scores). We mention this here to shed light on similarities and differences between the categorical and continuous analyses, and why it may be important to control for the other dimensions in the continuous analyses.

**Figure S2.** Distribution of Continuous Attachment Scores for Each Attachment Classification Group.


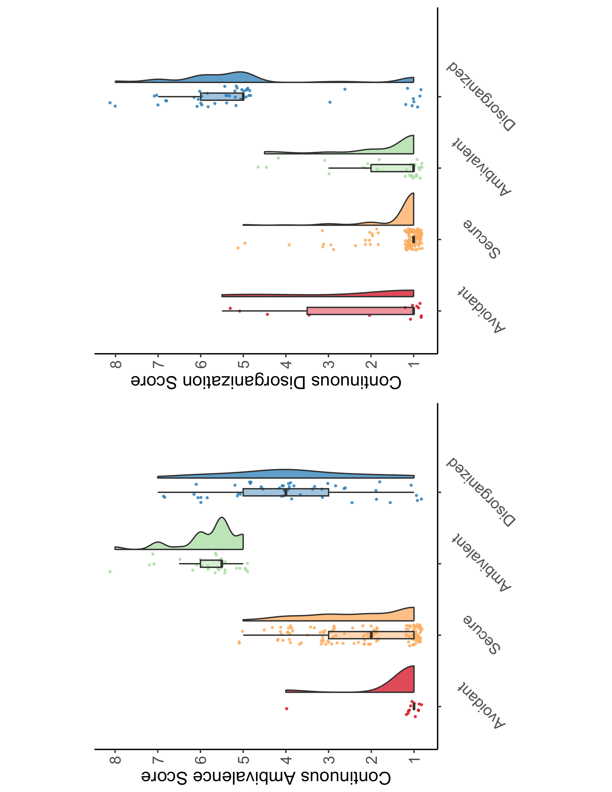

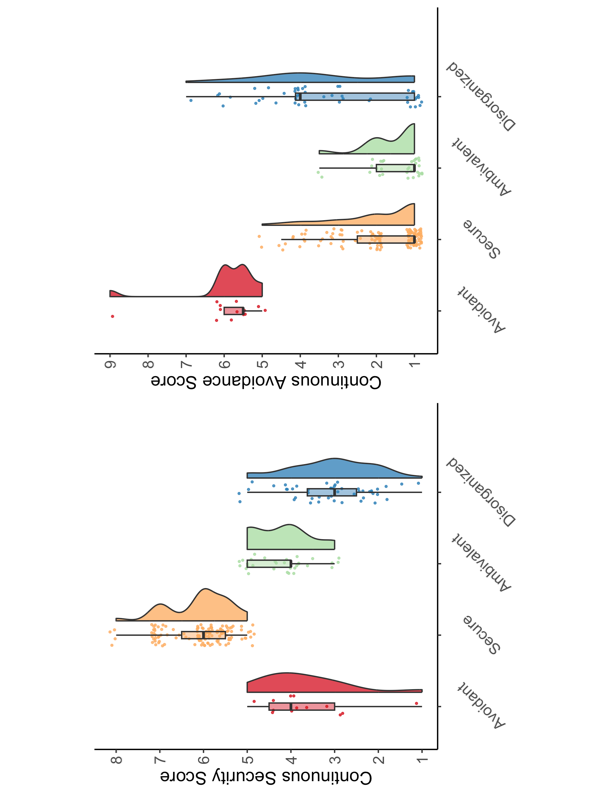


**Figure S3.** Comparison of Children’s Categorical Attachment Labels with Their Scores on the Continuous Attachment Dimensions.

**

**Johnson-Neyman Analysis**

In addition to the simple slope analyses reported in the main text, we used the Johnson-Neyman Technique (5) to identify thresholds of maternal sensitivity beyond which the two child *OPRM1* genotype groups significantly differed in the log odds of being classified ambivalent vs. secure. This analysis showed that, among children whose mothers exhibited lower levels of sensitivity (≥2 standard deviations below the mean), G allele carriers were more likely to be classified as ambivalent (vs. secure) relative to AA homozygotes. Additionally, G allele carriers were less likely to be classified as ambivalent (vs. secure) among children whose mothers were 1.3 standard deviations or higher above the mean in maternal sensitivity. This finding is illustrated in **Figure S4**.

**Figure S4.** Johnson-Neyman Plot Depicting Regions of Significance for the Child *OPRM1* Genotype by Maternal Sensitivity Interaction Predicting Child Ambivalence


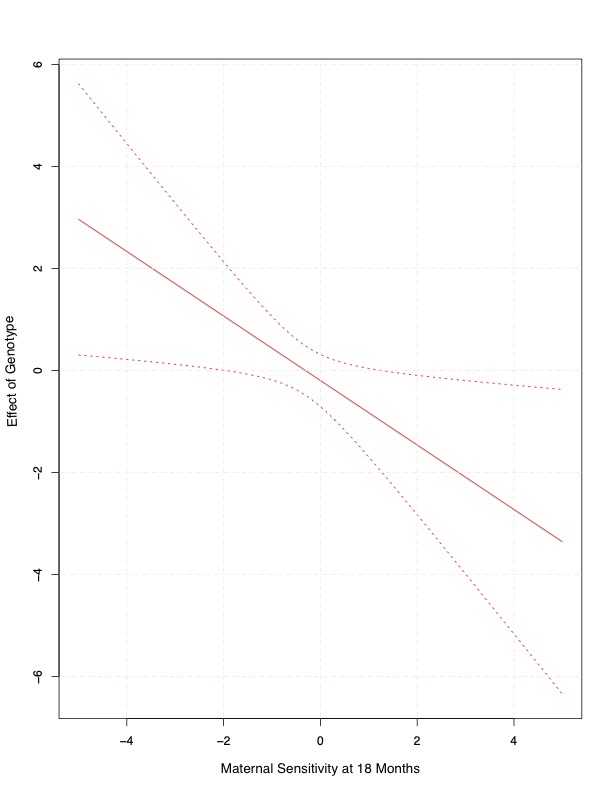


*Note*. The X-axis depicts the continuous range of standardized maternal sensitivity scores. The Y-axis depicts the continuous range of values for the adjusted effect of child genotype on the log odds of being classified as ambivalent (vs. secure). The straight, red line represents values of the adjusted child genotype effect. The curved lines represent 95% confidence bands around the adjusted effect of child genotype on ambivalence; note that the effect is significant in regions where the confidence bands do not include zero.

**References**

1. Cassidy J, Marvin RS (1992): *Attachment Organization in Preschool Children: Procedures and Coding Manual*. University of Virginia.

2. Moss E, Cyr C, Bureau JF, Tarabulsy GM, Dubois-Comtois K (2005): Stability of attachment during the preschool period. *Dev Psychol* 41: 773–783.

3. Deneault AA, Bureau JF, Yurkowski K, Moss E (2020): Validation of the Preschool Attachment Rating Scales with child-mother and child-father dyads. *Attach Hum Dev* 22: 491–513.

4. Moss E, Lecompte V, Bureau J-F (2015): *Preschool and Early School-Age Attachment Rating Scales (PARS)*. University of Quebec, Montreal.

5. Johnson PO, Fay LC (1950): The Johnson-Neyman Technique, its theory and application. *Psychometrika* 15: 349–367.

1. There were no significant effects for the avoidant or disorganization dimensions (all *p*s > .10); for the security dimension there was no effect of child *OPRM1* genotype, or maternal sensitivity (p=.11), but there was a significant child genotype x maternal sensitivity interaction *b*=-0.15, *p*=.042 (95% CI: -0.29, -0.01), such that maternal sensitivity at 18 months was positively associated with security among children homozygotic for the A allele, *b*=0.27, *p*<.001, but not among G allele carriers, *b*=-0.03, *p*=.818. [↑](#footnote-ref-1)
2. Again, there were no significant effects for the avoidant or disorganization dimensions; the aforementioned child genotype x maternal sensitivity interaction predicting security was non-significant (*p*=.083), with the other attachment dimensions in the model. [↑](#footnote-ref-2)
